# ErbB3 drives mammary epithelial survival and differentiation during pregnancy and lactation

**DOI:** 10.1186/s13058-017-0893-7

**Published:** 2017-09-08

**Authors:** Michelle M. Williams, David B. Vaught, Meghan Morrison Joly, Donna J. Hicks, Violeta Sanchez, Philip Owens, Bushra Rahman, David L. Elion, Justin M. Balko, Rebecca S. Cook

**Affiliations:** 1 Department of Cancer Biology, Vanderbilt University School of Medicine, 2220 Pierce Avenue, Rm 749 Preston Research Building, Nashville, TN 37232 USA; 2 Department of Medicine, Vanderbilt University Medical Center, Nashville, TN 37232 USA

**Keywords:** ErbB3, ErbB4, STAT5A, Jak2, Lactation, Mammary gland development, Prolactin, Alveolar mammary epithelial cell, PI3 kinase, Akt

## Abstract

**Background:**

During pregnancy, as the mammary gland prepares for synthesis and delivery of milk to newborns, a luminal mammary epithelial cell (MEC) subpopulation proliferates rapidly in response to systemic hormonal cues that activate STAT5A. While the receptor tyrosine kinase ErbB4 is required for STAT5A activation in MECs during pregnancy, it is unclear how ErbB3, a heterodimeric partner of ErbB4 and activator of phosphatidyl inositol-3 kinase (PI3K) signaling, contributes to lactogenic expansion of the mammary gland.

**Methods:**

We assessed mRNA expression levels by expression microarray of mouse mammary glands harvested throughout pregnancy and lactation. To study the role of ErbB3 in mammary gland lactogenesis, we used transgenic mice expressing WAP-driven Cre recombinase to generate a mouse model in which conditional ErbB3 ablation occurred specifically in alveolar mammary epithelial cells (aMECs).

**Results:**

Profiling of RNA from mouse MECs isolated throughout pregnancy revealed robust *Erbb3* induction during mid-to-late pregnancy, a time point when aMECs proliferate rapidly and undergo differentiation to support milk production. Litters nursed by *ErbB3* ^*KO*^ dams weighed significantly less when compared to litters nursed by *ErbB3* ^*WT*^ dams. Further analysis revealed substantially reduced epithelial content, decreased aMEC proliferation, and increased aMEC cell death during late pregnancy. Consistent with the potent ability of ErbB3 to activate cell survival through the PI3K/Akt pathway, we found impaired Akt phosphorylation in *ErbB3* ^*KO*^ samples, as well as impaired expression of STAT5A, a master regulator of lactogenesis. Constitutively active Akt rescued cell survival in ErbB3-depleted aMECs, but failed to restore STAT5A expression or activity. Interestingly, defects in growth and survival of *ErbB3* ^*KO*^ aMECs as well as Akt phosphorylation, STAT5A activity, and expression of milk-encoding genes observed in *ErbB3* ^*KO*^ MECs progressively improved between late pregnancy and lactation day 5. We found a compensatory upregulation of ErbB4 activity in *ErbB3* ^*KO*^ mammary glands. Enforced ErbB4 expression alleviated the consequences of ErbB3 ablation in aMECs, while combined ablation of both ErbB3 and ErbB4 exaggerated the phenotype.

**Conclusions:**

These studies demonstrate that ErbB3, like ErbB4, enhances lactogenic expansion and differentiation of the mammary gland during pregnancy, through activation of Akt and STAT5A, two targets crucial for lactation.

**Electronic supplementary material:**

The online version of this article (doi:10.1186/s13058-017-0893-7) contains supplementary material, which is available to authorized users.

## Background

The orchestrated development of the mammary gland requires several molecularly controlled, evolutionarily conserved, and temporally distinct processes [1], occurring to a large extent in adolescents and adults of mammalian species. The mammary gland is comprised of several distinct stem cell, progenitor cell, and mature mammary epithelial cell (MEC) subpopulations that each perform a specialized function at distinct developmental stages [2–7]. During pregnancy, as the mammary gland prepares for synthesis and delivery of milk to newborns, an apically located (i.e., luminal) MEC subpopulation proliferates rapidly in response to systemic hormonal cues such as progesterone and prolactin (PRL), as well as locally derived cues such as neuregulin (NRG, also known as heregulin). At parturition, these milk-producing MECs, referred to as alveolar MECs (aMECs), undergo terminal differentiation for milk synthesis and delivery [8]. This entire process is referred to as lactogenesis, a fundamental requirement for the survival of all mammalian species [9–11].

Several studies demonstrated that PRL-mediated milk production requires signaling through a molecular axis involving PRL receptor (PRLR)-mediated activation of the intracellular tyrosine kinase (TK) Janus kinase 2 (Jak2), which phosphorylates the transcription factor signal transducer and activator of transcription 5A (STAT5A), an obligate transactivator of several milk protein-encoding genes, including *Csn2* (the gene encoding β-casein) [8, 12–16]. Genetically engineered mouse models (GEMMs) confirmed that the PRL/PRLR/Jak2/STAT5A signaling axis is crucial for lactogenesis [17–19].

Although STAT5A is potently activated by PRLR/JAK2 signaling, STAT5A also forms complexes with other receptors in MECs [20, 21]. Among these is the receptor tyrosine kinase (RTK) ErbB4 [22–24], a member of the epidermal growth factor receptor (EGFR) RTK family, comprised of EGFR, HER2/ErbB2, HER3/ErbB3, and HER4/ErbB4. Interestingly, ErbB4 activity in the mammary gland, specifically in luminal aMECs, peaks during pregnancy and lactation [25], similar to what is seen for STAT5A. Multiple GEMMs of ErbB4 ablation in the mammary epithelium each display reduced expansion of alveolar structures during pregnancy, decreased terminal aMEC differentiation, impaired activation of STAT5A, lactation defects [20, 26, 27], and phenocopying of the effects of PRL, PRLR, JAK2, or STAT5A loss. Conversely, increased ErbB4 kinase signaling activates STAT5A, even in the absence of pregnancy-related hormones [28], confirming the role of ErbB4 in STAT5A-mediated lactogenesis.

ErbB4 is ligand activated by NRG family members (NRG1–4), and by certain EGF-like family members (heparin-binding EGF (HB-EGF), betacellulin (BTC), and epiregulin). *NRG1* expression by alveolar basal/myoepithelial MECs is induced at mid-gestation in response to p63, a master regulator of transcriptional programs directing cell fate. NRG initiates proliferation of adjacent aMECs through ErbB4/STAT5A activation [23]. Similar to what was seen with ErbB4 loss, mouse models lacking NRG1α or NRG1β expression [29], NRG bioavailability [23], or HB-EGF bioavailability [30] suffered decreased aMEC expansion during pregnancy. Conversely, NRG1-loaded slow release pellets implanted into mouse mammary glands induced precocious lactogenesis in nonpregnant mice [31]. The NRG ligands also bind to ErbB3, inducing ErbB3 heterodimerization with other EGFR family receptors, and indeed with a growing list of heterologous RTKs [32, 33]. Ligand-activated ErbB3 has been described as lacking intrinsic kinase activity [34], but once phosphorylated by a heterodimeric partner it potently stimulates cell survival signaling [35]. The role of ErbB3 in MEC cell survival is illustrated by distinct models of impaired ErbB3 signaling in the mammary epithelium during puberty, each causing impaired cell survival of ductal MECs and decreased lengthening of mammary duct.

The ability of ErbB3 to enhance cell survival is explained largely in part by its six binding sites for the p85 regulatory subunit of phosphatidyl inositol-3 kinase (PI3K) [24], more than any other RTK. When phosphorylated, ErbB3 interacts with p85 to promote PI3K activity, which generates the second messenger phosphoinositol-(3,4,5)-trisphosphate (PIP3), causing Akt recruitment, Akt phosphorylation (via PDK1 [36] and mTORC2 [37], which also are recruited by PIP3), and Akt kinase activation. Akt sits at the apex of a signaling cascade that supports cell survival, growth, metabolism, and cell cycle progression [38]. Several lines of evidence demonstrate that PI3K/Akt signaling supports lactogenesis of aMECs through cell survival. For example, models in which Akt signaling was impaired caused aMEC apoptosis, decreasing the capacity for lactogenic alveolar expansion during pregnancy, and causing premature mammary gland involution at parturition [39–42], while models of increased PI3K/Akt signaling, for example, through expression of myristoylated Akt1 (Akt^myr^) in the mammary epithelium, delayed the onset of postlactational cell death upon weaning [43–45]. Given that ErbB3 potently activates PI3K/Akt signaling, it is possible that ErbB3 may be required for lactogenic aMEC expansion during pregnancy. However, its role in aMECs is not yet clear, as two mouse models of ErbB3 ablation have produced disparate results. Although lactogenic aMEC expansion during pregnancy occurred normally in embryonic mammary bud transplants from a classical model of ErbB3 ablation to wild-type recipients [46], a knock-in model eliminating each of the ErbB3 p85-PI3K binding motifs within an otherwise intact ErbB3 reduced aMEC expansion during pregnancy, causing increased aMEC cell death and accelerated involution [47].

We used a mouse model of ErbB3 ablation specifically from aMECs, finding delayed aMEC expansion due to decreased Akt-dependent survival of cytokeratin 8 (CK8)-positive aMECs, and decreased STAT5A-mediated expression of milk-encoding genes. Restoration of Akt signaling rescued survival of ErbB3-depleted aMECs, but did not rescue STAT5A induction or milk protein expression. However, signaling through both Akt and STAT5A were rescued by ErbB4 overexpression. In vivo, compensatory ErbB4 upregulation in ErbB3-deficient MECs dampened the impact of ErbB3 ablation, allowing lactation to proceed, albeit in a delayed fashion, through the expansion of CK8^+^/CK5^+^ double-positive aMECs. These studies highlight the key roles played by ErbB3 and ErbB4 in establishing and maintaining milk production.

## Methods

### Mice

All animal husbandry and experiments were performed in accordance with protocols approved by the Vanderbilt University Institutional Animal Care Committee using humane procedures. *ErbB3* ^*FL*^ [48] and *WAPi-Cre* [49] mice have been described previously. All mice were inbred to, or generated on, the Friend Virus B-type (FVB) background. Mouse experiments were approved by the Vanderbilt Institutional Animal Care and Use Committee. Female virgin mice were bred at 10–12 weeks of age to WT FVB male mice. Mating pairs were separated upon identification of a semen plug, indicating 0.5 days post coitus (d.p.c.). Parturition indicated lactation day 0 (L0), and litters were normalized to eight pups per litter.

### Histological analysis

Right #4 inguinal mammary glands were formalin fixed and paraffin embedded, and sections (5 μm) were stained with hematoxylin and eosin. In-situ terminal dUTP nick end-labeling (TUNEL) analysis was performed on paraffin-embedded sections using the ApopTag kit (Millipore). Immunohistochemistry (IHC) on paraffin-embedded sections was performed using the following antibodies as described previously [50]: ErbB3 (C-17; Santa Cruz Biotechnology), Ki67 (Santa Cruz Biotechnology), P-Akt S473 (Cell Signaling Technologies), P-STAT5A/B (Neomarkers), and P-ErbB4 Tyr1056 (Cell Signaling Technologies). Immunodetection was performed using the Vectastain kit (Vector Laboratories) according to the manufacturer’s instructions. Immunofluorescence staining was performed with primary and secondary antibodies diluted in 12% Fraction-V BSA (Pierce) and slides were mounted in SlowFade mounting medium containing DAPI (Invitrogen). All fluorescent secondary antibodies were highly cross-adsorbed, produced in goat, and used at a dilution of 1:200 for 20 min (Molecular Probes). Primary antibodies used were CK5 (10956, 1:500; Covance/Biolegend), CK8/18 (1:500; Fitzgerald Industries International), and ErbB3 (c-17, 1:200; Santa Cruz Biotechnology).

### Cell culture

HC11 cells [51] were cultured in DMEM: F12 (1:1) medium supplemented with 10% fetal bovine serum (Life Science), insulin (5 μg/ml) and dexamethasone (10 μg/ml) (Sigma-Aldrich) and human EGF (5 ng/ml; R&D Systems). To induce differentiation, cells were serum and EGF-starved for 24 h, and then treated with 5 μg/ml mouse PRL (Preprotech). In some cases, cells were treated with Nrg1b (EGF-like domain) (R&D Systems), neratinib, AZD6244, or BKM120 (all from SelleckChem). Where indicated, single-cell suspensions (5 × 10^5^ cells) were infected with 10^6^ pfu/ml of the adenoviral particles adenoviral myristoylated Akt1 (Ad.Akt^myr^), adenoviral ErbB4 (Ad.ErbB4), and adenoviral green fluorescent protein (Ad.GFP), from Vector Biolabs. Knockdown of ErbB3 in HC11 cells using siRNA sequences was performed using 5 μM siRNA against ErbB3 (SASI_Mm01_0031804, SASI_Mm01_0031805, and SASI_Mm01_0031806; Sigma-Aldrich) or a scrambled sequence (siScr) transfected with Lipofectamine 2000 (Invitrogen). Stable knockdown of ErbB3 was achieved by lentiviral transduction of LVP158 (Gentarget, Inc.) HC11 cells were transduced with lentiviral particles for 48 h prior to puromycin selection. Pooled clones of puromycin-resistant cells were expanded for analysis of ErbB3 knockdown prior to experimentation.

### Western blot analysis

Mammary glands and cells were homogenized in ice-cold 50 mM Tris (pH 7.4), 100 mM NaF, 120 mM NaCl, 0.5% Nonidet P-40, 100 μM Na_3_VO_4_, and 1× protease inhibitor mixture (Roche), sonicated for 10 s on ice, and cleared by centrifugation at 4 °C, 13,000 × *g* for 20 min. Protein concentration was determined using the bicinchoninic acid assay (Pierce). Proteins were separated by SDS/PAGE and transferred to nitrocellulose membranes. Membranes were blocked in 3% gelatin in TBS-T (Tris-buffered saline, 0.1% Tween-20) for 1 h, incubated in primary antibody in 3% gelatin overnight at 4 °C, washed with TBS-T, incubated in HRP-conjugated anti-rabbit (sc-2375, 1:10,000; Santa Cruz Biotechnology) or anti-mouse IgG (ab97240, 1:5000; Abcam), washed with TBS-T, and then developed using ECL substrate (Pierce). The following primary antibodies were used: P-ErbB3 (sc-135654, 1:500; Santa Cruz Biotechnology), P-MAPK/Akt/S6/Rab11 cocktail (1:1000; Cell Signaling Technologies), total Akt (C67E7, 1:1000; Cell Signaling Technologies), actin (1:2000; Cell Signaling), STAT5A and P-STAT5A/B (93585 at 1:500 and C11C5 at 1:500, respectively; Cell Signaling Technologies), and cleaved caspase 3 (D175, 1:500; Cell Signaling Technologies).

### Gene expression analysis

Total RNA was harvested from cells and mammary glands using RNeasy (Qiagen). RNA quality was assessed using the Agilent Analyzer. RNA (1 μg) was reverse transcribed (RT2 First Strand Kit; Qiagen). Mouse primers for *Stat5a*, *Erbb4*, *Erbb3*, *Csn2*, and *36B4* were purchased from SABiosciences (Qiagen). Primer sequences are as follows: m*ErbB3*, forward 5′-CGAGAACTGCACCCAAGG and reverse 5′-TCTGCTTGGCCTAAACAGTCT; m*Staa5*A, forward 5′-CGCCAGATGCAAGTGTTGTAT and reverse 5′-TCCTGGGGATTATCCAAGTCAAT; m*Csn2*, forward 5′-GGCACAGGTTGTTCAGGCTT and reverse 5′-AAGGAAGGGTGCTACTTGCTG; and m*Erbb4*, forward 5′-CCATGGACCGGACCTGC and reverse 3′-GCTCCCTGTAGGCCATCTGG.

## Results

### ErbB3 loss decreases expansion of the mammary epithelium during pregnancy

**Fig. 1:**
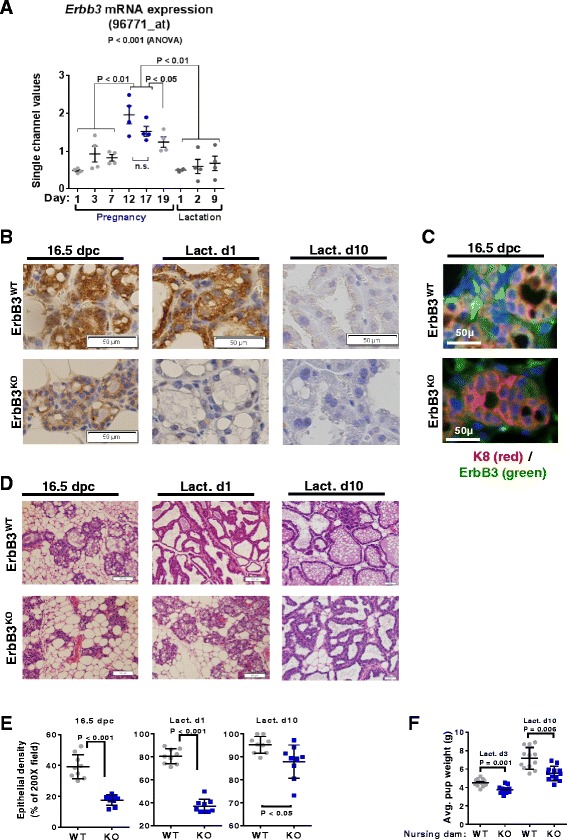
ErbB3 loss decreases expansion of the mammary epithelium during pregnancy. **a** *Erbb3* mRNA expression is maximal during late puberty in mice. Data and plot extracted and generated from the National Center for Biotechnology Information Gene Expression Omnibus dataset GDS2843 using the probe set 96771_at. Details of the study published in [60]. *Red columns* indicate the single channel transformed counts, while *blue data points* indicate the percentile rank of Erbb3 transcripts within the samples. **b**–**e** Mammary glands harvested from 12–15-week-old mice at 16 d.p.c. and L1. *N* = 9 per time point. Representative images are shown, original magnification is 400×. **b** IHC detection of ErbB3. **c** IF staining of K8 (*red*) and ErbB3 (*green*). Sections were counterstained with DAPI. **d** Histological sections stained with hematoxylin and eosin. **e** Quantitation of epithelial density in randomly selected 200× fields. Using digital images, epithelial areas were circumscribed to generate a closed polygon to generate an automated calculation of the polygon area. Each *data point* represents the average of three randomly chosen fields per sample; *midlines* are the average of all biological replicates ± SD. *P* values calculated using Student’s unpaired two-way *t* test. **f** Litters were normalized to eight pups each, then pups were weighed postnatally at days 3 and 10. Each *data point* represents the average pup weight of a single litter; *midlines* are the average of all litters within the group. *N* = 12 litters. Student’s unpaired, two-way *t* test. *dpc* days post coitus, *Lact d* lactation day (Color figure online)

We assessed *Erbb3* mRNA levels in expression microarray datasets derived from mouse mammary glands harvested at distinct developmental time points of pregnancy and lactation [52] (Fig. 1a), revealing relatively low *Erbb3* during early pregnancy (1–7 days post coitus (d.p.c.)) but approximately a 4-fold *Erbb3* upregulation at mid-pregnancy (12 d.p.c.). High *Erbb3* expression persisted through late pregnancy (18 d.p.c.) but decreased at parturition and lactation. We used immunohistochemistry (IHC) to identify the cell types of the mammary gland expressing ErbB3, confirming ErbB3 protein expression in developing alveoli (the milk-producing epithelial structures) (Fig. 1b). To investigate the impact of ErbB3 ablation from aMECs, we crossed mice harboring floxed *Erbb3* alleles (*Erbb3* ^*FL/FL*^) [48] with *WAPi-Cre* transgenic mice [49]. Compared to mammary glands from *ErbB3* ^*+/+*^ *WAP-Cre* mice (referred to here as *ErbB3* ^*WT*^), which expressed ErbB3 abundantly in aMECs at 16.5 d.p.c. and at lactation day 1 (L1), mammary glands from *ErbB3* ^*FL/FL*^ *WAP-Cre* mice (referred to here as *ErbB3* ^*KO*^) expressed potently reduced levels of ErbB3 (Fig. 1b; Additional file 1: Figure S1). Immunofluorescent staining of mammary glands harvested at 16.5 d.p.c. confirmed that ErbB3-positive MECs were also positive for cytokeratin 8 (K8) in *ErbB3* ^*WT*^ samples (Fig. 1c; Additional file 1 Figure S2). These findings are consistent with previous reports that ErbB3 is expressed primarily in luminal, but not basal, populations of the mammary epithelium [48].

Cre-mediated ErbB3 ablation decreased pregnancy-induced expansion of alveolar epithelial structures in mammary glands (Fig. 1d) by approximately 4-fold at 16.5 d.p.c. (Fig. 1e). Although epithelial area remained diminished in *ErbB3* ^*KO*^ mammary glands through L10, the effect size between *ErbB3* ^*KO*^ and *ErbB3* ^*WT*^ diminished progressively. Consistent with the decreased epithelial density seen in *ErbB3* ^*KO*^ mammary glands, pups nursed by *ErbB3* ^*KO*^ dams weighed less than those nursed by *ErbB3* ^*WT*^ dams at L3 and L10 (Fig. 1f). These data are in agreement with a previous report showing that ErbB3 signaling supports lactogenic expansion during pregnancy [47], and demonstrate that ErbB3 supports mammary gland development during pregnancy.

### ErbB3 loss decreases proliferation and cell survival of luminal, but not basal, MECs during pregnancy

**Fig. 2:**
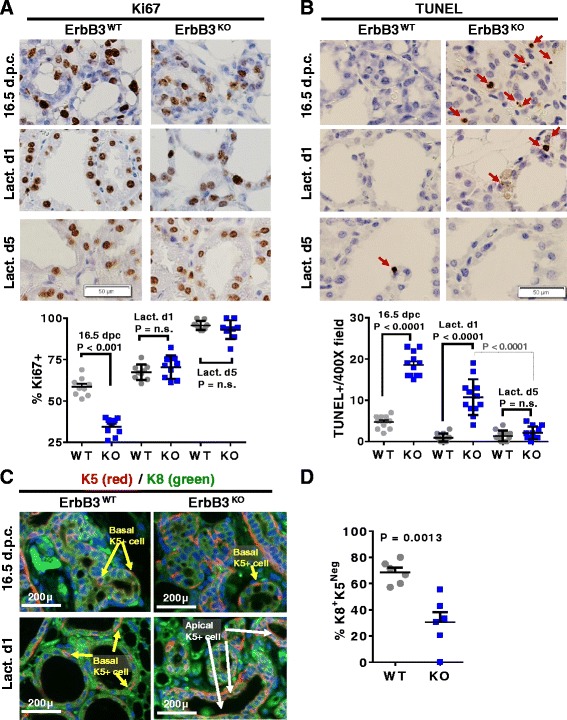
ErbB3 loss decreases proliferation and cell survival of luminal, but not basal, MECs during pregnancy. Mammary glands harvested from 12–15-week-old mice at 16.5 d.p.c., L1, and L5. Representative images are shown. Original magnification is 400×. *P* values calculated using Student’s unpaired two-way *t* test. **a** IHC detection of Ki67. *N* = 9. *Lower panel*: percentage of MECs that were Ki67^+^. Each *data point* represents the average of two random 400× fields from a single tumor; *midlines* are average of samples within the group ± SD. **b** TUNEL analysis, *N* = 12. Each *data point* represents the average of two random 400× field from a single tumor; *midlines* are the average of samples within the group ± SD. **c**. IF staining of K5 (*red*) and K8 (*green*). Sections were counterstained with DAPI. *Yellow arrows* indicate basal location of K5^+^ cells; *white arrows* indicate apically localized K5^+^ cells. **d** Quantitation of the percentage of MECs that were K8-positive/K5-negative. *N* = 5. Each *data point* represents the average value of three random 400× fields per sample; *midlines* are the average of all biological replicates ± SD. *dpc* days post coitus, *Lact d* lactation day, *TUNEL* terminal dUTP nick end-labeling (Color figure online)

To understand ErbB3-regulated events that contribute to lactogenic mammary gland development, we used Ki67 IHC as a measure of cell proliferation. These studies revealed decreased Ki67 staining in *ErbB3* ^*KO*^ aMECs at 16.5 d.p.c., as compared to *ErbB3* ^*WT*^ (Fig. 2a; Additional file 1: Figure S3). By L5, however, the percentage of Ki67^+^ aMECs was equal in *ErbB3* ^*KO*^ and *ErbB3* ^*WT*^ samples. Cell death of aMECs was measured in situ using terminal dUTP nick end-labeling (TUNEL) analysis, revealing markedly increased TUNEL^+^ cells in *ErbB3* ^*KO*^ mammary glands at 16.5 d.p.c. and L1, although no differences in the percentage of TUNEL^+^ MECs were seen by L5 (Fig. 2b; Additional file 1: Figure S4).

Immunofluorescent staining for K8 and cytokeratin 5 (K5) was used to discriminate between the K8^+^ populations of luminal MECs, the K5^+^ population of myoepithelial MECs and mammary stem cells, and K8^+^K5^+^ double-positive cells, which are thought to represent a bipotential MEC progenitor population and may contribute to the plasticity that exists between the distinct MEC lineages. Consistent with the reported histological architecture of the mouse mammary gland, *ErbB3* ^*WT*^ mammary glands harvested at L1 displayed basal localization of K5^+^ MECs and K8^+^K5^+^ double-positive MECs (yellow arrows, Fig. 2c) and apical localization of K8^+^ MECs. In contrast, *ErbB3* ^*KO*^ samples harvested at L1 harbored increased double-positive (K8^+^K5^+^) MECs, which were often localized to apical positions (white arrows, Fig. 2c). The increased numbers of K8^+^K5^+^ double-positive MECs appeared to occur at the expense of luminal (K8^+^) MECs, since K8^+^ MECs were decreased in *ErbB3* ^*KO*^ samples as compared to controls (Fig. 2d). This finding that ErbB3 depletion decreases the population of K8^+^ MECs is consistent with previous observations that ErbB3 loss from the mammary ductal epithelium caused a loss of K8^+^ luminal MECs, with a compensatory expansion of the K5^+^ population [48].

### ErbB3 drives MEC survival through the PI3K-to-Akt signaling pathway during pregnancy

**Fig. 3:**
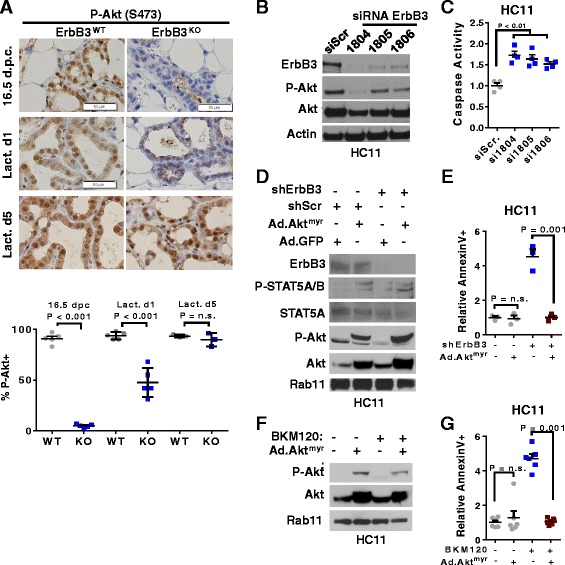
ErbB3 drives MEC survival through the PI3K-to-Akt signaling pathway during pregnancy. **a** Mammary glands harvested from 12–15-week-old mice at 16.5 d.p.c., L1, and L5, and stained using IHC for P-Akt Serine 473. Representative images are shown. Original magnification is 400×. *Lower panel*: percentage of MECs that were P^+^. Each *data point* represents the average of two random 400× fields from a single tumor; *midlines* are the average of samples within the group ± SD. *P* values calculated using Student’s unpaired two-way *t* test. *N* = 5. **b**, **c** HC11 cells harvested for analysis 72 h after transfection with three distinct siRNA sequences targeting ErbB3, or a scrambled siRNA sequence (siScr). **b** Western blot analysis using antibodies listed at the *left*. Representative images are shown, repeated three times. **c** Caspase 3/7 activity measured using Caspase-Glo, corrected for total protein content, and shown relative to the caspase 3/7 activity measured in cells transfected with siScr sequences. Experiments were assessed in triplicate and repeated four times. *Data points* are the average of the experimental triplicates; *midlines* are the average (± SD) of the biological replicates. Student’s unpaired *t* test. **d, e** HC11 cells stably transduced with lentivirus encoding ErbB3 shRNA (shErbB3) or shScr, then transduced with Ad.Akt^Myr^ or Ad.GFP, and harvested for analysis 48 h later. **d** Western blot analysis using antibodies listed at the *left*. Representative images are shown, repeated three times. **e** Cell cultures stained with Annexin V-FITC to detect apoptotic cells. Experiments were assessed in triplicate and repeated four times. *Data points* are the average of the experimental triplicates across experiments; *midlines* are the average (± SD) of the biological replicates. Student’s unpaired *t* test. **f**, **g** HC11 cells transduced with Ad.Akt^Myr^ or Ad.GFP. At 48 h post transduction, cells were treated with BKM120 (1 μM) for 6 h (**f**) or 16 h (**g**). **f** Western blot analysis using antibodies listed at the *left*. Representative images are shown, repeated three times. **g** Cell cultures stained with Annexin V-FITC to detect apoptotic cells as described in (**e**)**. h**, **i** HC11 cells treated with BKM120 (1 μM) or AZD6244 (1 μM) for 6 h (**h**) or 16 h (**i**). **h** Western blot analysis using antibodies listed at the *left*. Representative images are shown, repeated three times. **i** Cell cultures stained with Annexin V-FITC to detect apoptotic cells as described in (**e**). *dpc* days post coitus, *Lact d* lactation day

Given that ErbB3 is a potent driver of PI3K/Akt signaling in ductal luminal MECs [48] and in luminal breast cancers [53], we examined P-Akt in mammary glands harvested at 16.5 d.p.c., L1, and L5. Consistent with previous reports of Akt signaling during lactogenic differentiation, we found abundant Akt phosphorylation in alveolar structures of the mammary glands harvested from *ErbB3* ^*WT*^ mice at each time point (Fig. 3a; Additional file 1: Figure S5). In contrast, Akt phosphorylation was significantly diminished at 16.5 d.p.c. in *ErbB3* ^*KO*^ samples, but began to recover by L1 and we observed full restoration of P-Akt by L5.

To determine the role of Akt signaling as an ErbB3 effector driving MEC survival during lactogenesis, we utilized HC11 cells, an immortalized aMEC cell line harvested during pregnancy from a Balb/C mouse. Although immortalized, these cells retain their ability to undergo lactogenic differentiation in culture [51], including PRL-inducible expression of milk proteins and formation of fluid-filled acini. We knocked down ErbB3 in HC11 cells using three distinct siRNA sequences, each of which potently reduced ErbB3 protein levels (Fig. 3b). Similar to what was seen in mouse mammary glands in vivo, loss of ErbB3 in HC11 cells decreased Akt phosphorylation, and increased activation of caspases 3 and 7 (Fig. 3c), irreversible molecular switches that activate programmed cell death. Next, we generated lentiviral vectors for shRNA-mediated stable knockdown of ErbB3 in HC11 cells, which decreased ErbB3 protein expression in pooled selection-resistant clones as compared to HC11 cells expressing a scrambled nontargeting shRNA (shScr; Fig. 3d). ErbB3 knockdown efficiently reduced phosphorylation of Akt. We restored Akt activity in HC11-shErbB3 using adenoviral expression of Akt^myr^ (Ad. Akt^myr^), which rescued Akt phosphorylation in HC11-shErbB3 cells. ErbB3 knockdown by shRNA increased HC11 apoptosis over what was seen in shScr-expressing cells, as measured by Annexin V staining (Fig. 3e). However, Ad.Akt^myr^ restored survival of HC11-shErbB3 cells, suggesting that ErbB3 utilizes Akt signaling to drive aMEC survival.

PI3K signaling activates Akt in numerous cell types, including ductal luminal MECs [47, 48]. To determine whether PI3K is required to activate Akt in aMECs, we used the pan-PI3K p110 subunit kinase inhibitor BKM120 to block PI3K signaling in HC11 cells, which impaired Akt phosphorylation (Fig. 3f). Ad.Akt^myr^ restored Akt phosphorylation in BKM120-treated cells. Similar to what was seen upon ErbB3 depletion, PI3K inhibition using BKM120 potently increased HC11 apoptosis (Fig. 3g). Ad.Akt^myr^ decreased HC11 cell apoptosis following treatment with BKM120. Since ErbB3 signaling also activates the mitogen activated protein kinase (MAPK) pathway in ductal luminal MECs during puberty [48], we assessed the impact of MAPK inhibition on HC11 cell survival by treating cells with the MEK inhibitor AZD6244. MEK inhibition blocked Erk phosphorylation, as expected (Additional file 1: Figure S6A). In contrast to what was seen with PI3K inhibition, MEK inhibition did not induce apoptosis in HC11 cells (Additional file 1: Figure S6B), suggesting that aMECs rely on ErbB3 signaling to drive PI3K/Akt-mediated cell survival during lactogenic expansion.

### Decreased STAT5-dependent differentiation of ErbB3-deficient aMECs

**Fig. 4:**
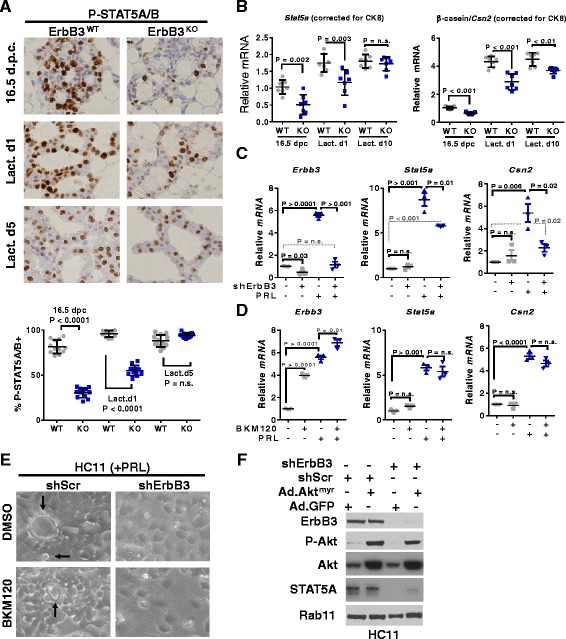
Decreased STAT5-dependent differentiation of ErbB3-deficient aMECs. **a**, **b** Mammary glands harvested from 12–15-week-old mice at 16.5 d.p.c., L1, and L5. **a** Samples stained using IHC for P-STAT5A/B. Representative images are shown, and original magnification is 400×. *Lower panel*: percentage of MECs that were P-STAT5A/B^+^. Each *data point* represents the average of two random 400× fields from a single tumor; *midlines* are the average of samples within the group ± SD. *P* values calculated using Student’s unpaired two-way *t* test. *N* = 12. **b** Whole tissue RNA assessed by RT-qPCR for *Stat5a*, *Csn2*, and *Ck8*. Relative transcript levels calculated using the ΔΔCt method, internally correcting for *36B4* gene expression. Values are shown relative to the average value obtained for ErbB3WT samples harvested at 16.5 d.p.c. Each sample was assessed in triplicate, with the average triplicate value shown by a single *data point. N* = 7 per time point. **c**–**e** HC11-shErbB3 and HC11-shScr cells (**c**, **e**) and parental HC11 cells (**d**) were serum and EGF-starved for 24 h, followed by treatment with PRL, with or without BKM120 (1 μM) for 24 h. **c**, **d** Whole cell RNA assessed by RT-qPCR for expression of *Stat5a* and *Csn2* and quantitated as described for (**b**). **e** Representative image of serum/EGF-starved cells treated for 24 h with PRL, revealing fluid-filled acini (*black arrows*) in ShScr cells, but not in shErbB3 cells. **f** HC11-shErbB3 and HC11-shScr cells transduced with Ad.Akt^Myr^ or Ad.GFP. At 48 h after transduction, cells were serum and EGF-starved, then treated with PRL for 24 h, and assessed by western blot analysis using antibodies shown at the *left. dpc* days post coitus, *Lact d* lactation day, *PRL* prolactin (Color figure online)

Several reports describe the reciprocal relationship between Akt activation and STAT5A activation in the mammary gland [41, 42]. Importantly, STAT5A is a transcription factor that drives aMEC expansion and lactogenic differentiation during pregnancy [18, 19, 54]. Because Akt activity was profoundly attenuated in *ErbB3* ^*KO*^ aMECs during pregnancy, we assessed STAT5 activity in *ErbB3* ^*KO*^ mammary glands by examining STAT5 phosphorylation. We found an abundance of cells expressing P-STAT5A/B in mammary glands harvested from *ErbB3* ^*WT*^ mice at 16.5 d.p.c., L1, and L5 (Fig. 4a; Additional file 1: Figure S7). In contrast, few aMECs expressed P-STAT5A/B in *ErbB3* ^*KO*^ mammary glands at 16.5 d.p.c. Although the percentage of aMECs expressing P-STAT5A/B remained significantly reduced in *ErbB3* ^*KO*^ mammary glands at L1, the percentage of cells expressing P-STAT5A/B in *ErbB3* ^*KO*^ mammary glands at L5 was similar to what was seen in stage-matched *ErbB3* ^*WT*^ samples, analogous to the temporal recovery of P-Akt in *ErbB3* ^*KO*^ samples. We measured *Stat5a* gene expression in whole mammary glands. To account for reduced input of luminal MECs in *ErbB3* ^*KO*^ samples, all values were corrected for expression of *Ck8*, the gene encoding K8. These results revealed reduced *Stat5a* mRNA expression in *ErbB3* ^*KO*^ samples at 16.5 d.p.c. and L1 (Fig. 4b), although *Stat5a* expression was similar in *ErbB3* ^*KO*^ and *ErbB3* ^*WT*^ samples at L10. Because STAT5A binds to the promoters of several prominent milk protein encoding genes, including *Csn2* (which encodes β-casein), to drive their expression during lactogenesis and lactation, we examined gene expression of *Csn2*. Similar to what was seen with *Stat5a* expression, *Csn2* was decreased in *ErbB3* ^*KO*^ samples at 16.5 d.p.c., L1, and L10, although *Csn2* levels progressively increased throughout lactation.

We used HC11-shErbB3 and HC11-shScr cells to investigate ErbB3-inducible signaling pathways that regulate lactogenic differentiation. Serum and EGF-starved HC11-shScr cells were treated with PRL for 24 h to initiate lactogenic differentiation, as described previously [51], which upregulated *Erbb3*, *Stat5a*, and *Csn2* transcripts (Fig. 4c), similar to the *Erbb3* induction in mammary glands seen during mid-pregnancy (Fig. 1a) when systemic hormones induce lactogenic expansion of the mammary gland. In contrast, HC11-shErbB3 cells cultured under these same conditions failed to induce *Erbb3*, *Stat5a*, and *Csn2* (Fig. 4c).

We next tested the role of PI3K signaling in lactogenic HC11 differentiation, using the PI3K inhibitor BKM120. Serum and EGF-starved HC11 cells cultured for 24 h with BKM120 revealed upregulation of *Erbb3* mRNA (Fig. 4d), consistent with previous observations that PI3K inhibition causes increased *Erbb3* gene expression in breast, lung, and intestinal tumor cells [56, 57]. In fact, BKM120 enhanced PRL-mediated induction of *Erbb3* in HC11 cells. Further, PI3K inhibition using BKM120 did not interfere with PRL-mediated induction of *Stat5a* or *Csn2* transcripts. While PRL-treated HC11-shScr cells generated fluid-filled acini, a morphological feature consistent with HC11 cell differentiation [12, 21, 28], HC11-shErbB3 cells were unable to generate these fluid-filled acini (Fig. 4e). However, PI3K inhibition in HC11-shScr cells using BKM120 did not impair formation of fluid-filled acini, although these acini were smaller than what was seen in control samples, consistent with the qualitatively smaller size of the BKM120 cells adjacent to the acini. Further, Akt^myr^ expression in HC11-shErbB3 cells only modestly rescued STAT5A protein expression under basal conditions (Fig. 4f). These results suggest that, while ErbB3 signaling is required for induction of *Stat5a* and *Csn2*, PI3K/Akt signaling is not.

### ErbB4 loss compensates for ErbB3 loss in aMECs during pregnancy and lactation

**Fig. 5:**
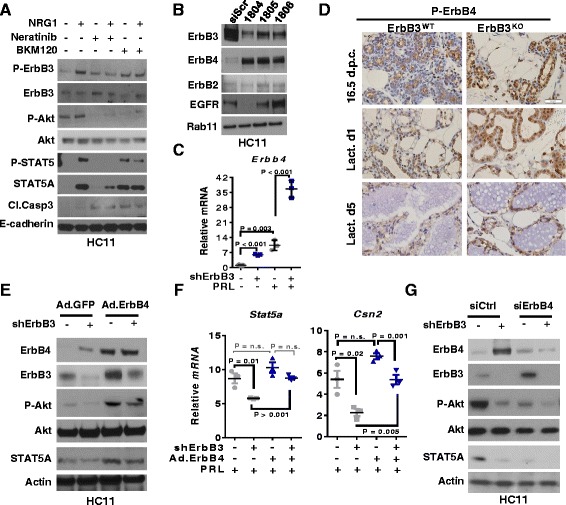
ErbB4 loss compensates for ErbB3 loss in aMECs during pregnancy and lactation. **a** HC11 cells serum and EGF-starved for 48 h were treated with PRL for 24 h, in the presence of NRG1 (2 ng/ml), neratinib (250 nM), and/or BKM120 (500 nM) as indicated. Whole cell lysates assessed by western blot analysis for proteins indicated at the *left*. **b** HC11 cells harvested for analysis 72 h after transfection with three distinct siRNA sequences targeting ErbB3, or a scrambled siRNA sequence (siScr). Western blot analysis using antibodies listed at the *left*. Representative images are shown, repeated three times. **c** HC11-shErbB3 and HC11-shScr cells serum and EGF-starved for 24 h, followed by treatment ± PRL for 24 h. Whole cell RNA assessed by RT-qPCR for expression of *Erbb4* and quantitated as described for Fig. 4b. **d** Samples stained using IHC for P-ErbB4 Tyr1056. Representative images are shown, and original magnification is 400×. *N* = 12. **e**, **f** HC11-shErbB3 and HC11-shScr cells transduced with Ad.ErbB4 or Ad.GFP. At 48 h after transduction, cells were serum and EGF-starved, and then treated with PRL for 24 h. **e** Cells assessed by western blot analysis using antibodies shown at the *left*. **f** Whole cell RNA assessed for expression of *Stat5a* and *Csn2* and quantitated as described in Fig. 4b. **g** HC11-shErbB3 and HC11-shScr cells were transfected with control siRNA sequences (*siCtrl*) or siRNA sequences targeting ErbB4 (*siErbB4*). At 72 h after transfection, cells were serum and EGF-starved, then treated with PRL for 24 h. Cells assessed by western blot analysis using antibodies shown at the *left. dpc* days post coitus, *Lact d* lactation day, *PRL* prolactin

ErbB3 heterodimerization is induced by the NRG ligands. We grew HC11 cells to confluence, then serum and EGF-starved the cells for 24 h and treated cells with PRL in the presence or absence of NRG1 (2 ng/ml), revealing increased P-ErbB3, total STAT5A, and P-STAT5A/B at 24 h (Fig. 5a). Treatment with the pan-ErbB family inhibitor neratinib (0.25 μM) blocked NRG1-induced phosphorylation of ErbB3 and STAT5A/B, and largely reduced NRG1-mediated total STAT5A induction. Neratinib treatment also blocked basal and NRG1-induced Akt phosphorylation, while increasing caspase 3 cleavage. Similarly, BKM120 treatment blocked basal and NRG1-induced Akt phosphorylation, and increased caspase 3 cleavage. However, levels of STAT5A and P-STAT5A/B remained elevated in BKM120-treated HC11 cells. Further, NRG1 treatment of HC11-shErbB3 cells for 24 h partially rescued the induction of cell death caused by ErbB3 ablation (Additional file 1: Figure S8). These data suggest that NRG-induced ErbB3 signaling potently induces both PI3K/Akt and STAT5A signaling pathways, but that the PI3K/Akt signaling may not be necessary for the induction of STAT5A during early aMEC expansion.

Because NRG ligands also bind to and activate ErbB4, a heterodimeric partner of ErbB3 that promotes lactogenic differentiation, we measured ErbB4 expression levels in HC11 cells expressing siRNA sequences against ErbB3 at 72 h post transfection, revealing increased expression of ErbB4 upon depletion of ErbB3 (Fig. 5b). These results were confirmed in serum and EGF-starved HC11-shErbB3 cells, revealing increased *Erbb4* gene expression levels over what was seen in HC11-shScr cells (Fig. 5c). PRL increased *Erbb4* expression in HC11-shScr cells, and to an even further extent in HC11-shErbB3 cells. These results support the idea that NRG-mediated signaling through ErbB4 may compensate for ErbB3 ablation, resulting in increased signaling though PI3K/Akt, and inducing expression and activity of STAT5A.

We tested this hypothesis first by assessing expression and activity of the Cyt-1 isoform of ErbB4. ErbB4 harbors two splice variations in its intracellular domain. The Cyt-1 isoform harbors a PI3K-binding site at Tyr 1056, which requires phosphorylation in order to support an interaction with PI3K. Importantly, the Cyt-1 isoform is a potent driver of lactogenic differentiation. In contrast, the Cyt-2 isoform lacks the PI3K binding motif, and is a less potent activator of lactogenic differentiation. IHC-mediated detection of P-ErbB4 (Tyr 1056) was used to detect the presence of activated ErbB4-Cyt1 in mammary gland sections, revealing increased P-ErbB4 in *ErbB3* ^*KO*^ mammary glands as compared to *ErbB3* ^*WT*^ mammary glands at late pregnancy and L1 (Fig. 5d).

Next, we overexpressed full-length ErbB4 (Cyt-1) in HC11-shErbB3 cells to determine whether ErbB4 upregulation might compensate for ErbB3 loss. Although stable knockdown of ErbB3 in HC11-shErbB3 cells resulted in ErbB4 upregulation, ErbB4 levels were increased much further upon transduction with adenoviral ErbB4 (Ad.ErbB4, Fig. 5e). ErbB4 overexpression in HC11-shErbB3 increased STAT5A and P-Akt expression, consistent with the idea that ErbB4 signaling supports PI3K signaling to Akt, and enhances lactogenic induction of STAT5A. Additionally, *Csn2* expression was reduced in HC11-shErbB3 cells transduced with Ad.GFP, but Ad.ErbB4 rescued *Csn2* levels in HC11-shErbB3 cells (Fig. 5f). Conversely, siRNA-mediated knockdown of ErbB4 caused ErbB3 upregulation in HC11-shControl cells (Fig. 5g). Despite robust ErbB3 expression in cells depleted of ErbB4, both P-Akt and STAT5A levels remained low, suggesting that ErbB3 and ErbB4 may operate as a functional heterodimeric unit to promote signaling that drives lactogenic differentiation. Further, elimination of NRG signaling through the combined knockdown of both ErbB3 and ErbB4 produced an even greater inhibitory effect on P-Akt and STAT5A. These findings suggest that the NRG receptors, ErbB3 and ErbB4, cooperatively enhance STAT5A and P-Akt signaling during lactogenic expansion and differentiation of the mouse mammary epithelium, and that loss of ErbB3 can be compensated for, at least partially, by increased signaling through ErbB4.

## Discussion

We demonstrate here, using aMEC-specific ErbB3 ablation, that ErbB3 engages two key molecular signaling pathways at mid-pregnancy in the mammary gland, STAT5A and PI3K/Akt, which regulate two nonoverlapping aspects of lactogenic development during mid-pregnancy. We show that STAT5 signaling is a critical driver of lactogenic differentiation downstream of ErbB3, while PI3K/Akt signaling downstream of ErbB3 is needed for survival of the rapidly expanding aMEC population during pregnancy. These findings are consistent with a study in which ErbB3-to-PI3K signaling was eliminated through knock-in of an ErbB3 mutant lacking all PI3K binding motifs [47]. Interestingly, previous studies confirm that ErbB3 is required in luminal progenitor populations [48], which may give rise to early alveolar progenitor MECs. Because the previous study used a systemic ErbB3 mutant knock-in, then it is possible that impaired ErbB3–PI3K signaling would have altered the luminal progenitor population of MECs prior to commitment to the alveolar lineage, indirectly decreasing the aMEC population, interfering with lactogenesis, and potentially confounding the results. The studies performed herein addressed this possibility, using a Cre-expressing model confined to the committed alveolar lineage [49], and thus sparing ErbB3 expression in any luminal progenitor populations that might not yet be committed to the alveolar lineage. However, the results shown here largely recapitulate what was seen with the systemic ErbB3 mutant knock-in model, supporting the notion that ErbB3 drives survival of luminal aMECs even after lineage specification.

We further distinguish our findings from those using the ErbB3 mutant knock-in model, as well as the ErbB3-null mammary bud transplantation model, by our observations that complete ErbB3 disruption leads to decreased expression and phosphorylation of STAT5A in aMECs. This phenotype may have been missed in the previous models, perhaps because the knock-in ErbB3 mutant remains capable of ligand-induced heterodimerization with other ErbB family members, which would be capable of NRG-mediated ErbB3–ErbB4 signaling, which was potentially sufficient to induce STAT5A upregulation. This hypothesis is supported by our observations here that ErbB4 can partially, but not fully, compensate for ErbB3 loss.

The PI3K/Akt pathway is a key signaling node for lactogenic expansion and differentiation of the luminal mammary epithelium. Numerous signaling pathways that regulate lactogenic development converge on PI3K/Akt, including the insulin-like growth factor 1 receptor (IGF1R), RANKL and RANK, integrins, and PRLR-to-JAK2-to-STAT5A pathways [8, 11, 14, 33, 44]. In fact, transgenic mice overexpressing Akt or expressing constitutively active Akt^myr^ in the mammary epithelium displayed increased aMEC survival, delaying the onset of MEC apoptosis at weaning [40, 41, 43, 45]. Although cell survival was not affected during pregnancy or lactation per se, increased activity of Akt aberrantly increased glucose uptake, glycolysis, and lipid production by aMECs, causing milk stasis and insufficient lactation [43], suggesting that Akt supports functions beyond cell survival in aMECs.

We show here that ErbB3 is expressed in mammary glands at mid-pregnancy, a time point when NRG ligands [23, 29, 31], ErbB4 [20, 26, 27], and STAT5 [15, 18, 19, 54] are induced. These findings suggest that ErbB3 may participate, perhaps as a heterodimeric partner of ErbB4, in the signaling cascade that activates STAT5A expression and phosphorylation, while also potently activating Akt phosphorylation at mid-pregnancy. This was confirmed in experiments in which ErbB4 upregulation rescued Akt and STAT5A dysregulation in response to ErbB3 ablation, whereas ErbB4 knock-down exacerbated the defects in Akt and STAT5A signaling caused by ErbB3 depletion (Fig. 5). This does not rule out the possibility that other EGFR family RTKs or ligand-activated PRLR might interact with ErbB3 and/or ErbB4 during lactogenesis, either as heterodimers or as multimeric signaling units. Because transgenic mice expressing a mammary-specific dominant-negative ErbB2 mutant have defective aMEC expansion and milk production [58], while mice lacking the EGFR ligands amphiregulin or EGF display reduced aMEC growth during pregnancy [59], it is possible that EGFR and/or ErbB2 might play a supporting role in aMEC expansion during pregnancy. This is an attractive hypothesis, given that ErbB4 upregulation occurred rapidly in *ErbB3* ^*KO*^ mammary glands, as early as 16.5 d.p.c., but restoration of STAT5A and P-Akt was not evident until lactation day 1. However, we did not find compensatory upregulation of ErbB2 expression in HC11 cells expressing shErbB3 (Additional file 1: Figure S9A) or in *ErbB3* ^*KO*^ mammary glands (Additional file 1: Figure S9B).

Because previous reports suggest that ErbB4 can exist within a physical complex with PRLR, leading to NRG-induced PRLR activation, and conversely PRL-dependent ErbB4 phosphorylation [12], it is possible that ligand-activated PRLR could trans-activate ErbB4, resulting in low levels of ErbB4 activity that may partially compensate for ErbB3 loss. Consistent with this idea, treatment of HC11 cells with PRL induced low levels of ErbB4, ErbB3, and STAT5A/B phosphorylation (Additional file 1: Figure S10A, B), suggesting some level of cross-talk between PRL and ErbB RTKs. However, PRL treatment did not induce Akt phosphorylation. Further, ErbB3 knockdown interfered with PRL-mediated STAT5A/B phosphorylation, suggesting an important role for ErbB3 in PRL-mediated STAT5A/B phosphorylation, at least in the setting of a 30-month PRL treatment.

The observation that ErbB4 upregulation partially alleviated the impact of ErbB3 loss upon Akt phosphorylation and cell survival is consistent with previous studies demonstrating that full-length ErbB4 (the Cyt-1 isoform) harbors a PI3K binding motif capable of PI3K activation when phosphorylated at Tyr-1056. Notably, we identified potent upregulation of P-ErbB4 Tyr-1056 in *ErbB3* ^*KO*^ mammary glands, Interestingly, inhibition of PI3K was unable to modulate STAT5A levels in HC11 cells, and restoration of Akt signaling in ErbB3-depleted HC11 was not sufficient to restore STAT5A expression, despite the previously described ability of Akt to enhance STAT5A stabilization and activity. Because restoration of Akt signaling rescued cell survival but did not rescue STAT5A expression, it is unlikely that selective death of STAT5A-expressing cells is the reason why STAT5A expression is low in the absence of ErbB3. The molecular mechanisms underlying decreased STAT5A expression remain unclear, and will require additional studies. However, we found that ErbB4 upregulation was capable of restoring STAT5A expression in the absence of ErbB3, highlighting the ability of ErbB3 and ErbB4 to compensate for one another. These observations are supported by our findings that the combined loss of both ErbB3 and ErbB4 further impaired Akt and STAT5A signaling in aMECs (Fig. 5f), thus supporting both cell survival (PI3K to Akt) and lineage commitment/differentiation (STAT5A).

## Conclusions

In summary, we have found that the NRG receptor ErbB3 supports lactogenic expansion of the milk-producing alveolar mammary epithelium during pregnancy, similar to what was found for the only other known NRG receptor, ErbB4. Given the importance of lactation for the survival of mammalian species, it may not be surprising that compensatory signaling mechanisms exist, ones that will ensure adequate lactation even if a single pathway is compromised. The important roles played by the NRG ligands and their two receptors in driving lactogenesis of the mammary epithelium during pregnancy are becoming increasingly evident. Although PRL is a known activator of STAT5 in the mammary gland, these data showing ErbB3-dependent induction of STAT5 expression, taken with previous data demonstrating ErbB4-mediated phosphorylation and activation of STAT5, suggest that NRGs produced locally in the mammary microenvironment enable PRL-mediated STAT5A activation, while at the same time promoting PI3K/Akt signaling, which together amplify, sustain, and differentiate this unique cell population on which newborn mammals depend.

## Additional file

Additional file 1: is **Figure S1** showing ErbB3 IHC of mammary glands harvested at 16.5 d.p.c. and L1, **Figure S2** showing immunofluorescence for ErbB3 and Cytokeratin-8 (K8) in mammary glands harvested at 16.5 d.p.c., **Figure S3** showing mammary glands harvested from 12–15-week-old mice at 16.5 d.p.c., L1 and L5, and stained using IHC for Ki67 (representative images, original magnification 400×), **Figure S4** showing mammary glands harvested from 12–15-week-old mice at 16.5 d.p.c. and L1, assessed by TUNEL analysis in situ (representative images, original magnification 400×), **Figure S5** showing P-Akt S473 IHC of mammary glands harvested from 12–15-week-old mice at 16.5 d.p.c., L1, and L5, and stained using IHC for P-Akt Serine 473 (representative images, original magnification 400×), **Figure S6** showing HC11 cells treated with BKM120 (1 μM) or AZD6244 (1 μM) for 4 h: **A** western blot analysis using antibodies listed at the *left* (representative images, repeated three times) and **B** cell cultures stained with Annexin V-FITC to detect apoptotic cells, and Annexin V^+^ cells quantitated in digital images (experiments assessed in duplicate and repeated three times; *data points* are the average of the experimental duplicates; *midlines* are the average (± SD) of the biological replicates; Student’s unpaired *t* test), **Figure S7** showing immunohistochemical detection of P-STAT5 A/B in mammary glands, **Figure S8** showing HC11 cells expressing shErbB3 or shScr serum and EGF-starved for 24 h in the presence or absence of NRG1β (2 ng/ml), then stained with Annexin V-FITC to detect apoptotic cells, and Annexin V^+^ cells quantitated in digital images (experiments assessed in duplicate and repeated three times; *data points* are the average of the experimental duplicates; *midlines* are the average (± SD) of the biological replicates; Student’s unpaired *t* test), **Figure S9** showing **A** HC11 cells expressing shErbB3 or shScr serum and EGF-starved for 24 h in the presence or absence of PRL, then whole RNA assessed by RT-qPCR for transcripts encoding ErbB2 and EGFR (experiments assessed in duplicate, repeated three times; *data points* are the average of the experimental duplicates; *midlines* are the average (± SD) of the biological replicates; Student’s unpaired *t* test) and **B**, **C** IHC used to visualize ErbB2 expression in mammary glands harvested at 16.5 d.p.c., L1, and L5 from uniparous *ErbB3* ^*WT*^ and *ErbB3* ^*KO*^ mice (panels in (**C**) identical to panels in (**B**), but include a wider field of view), and **Figure S10** showing **A** HC11 cells expressing shErbB3 or shScr serum and EGF-starved for 30 min in the presence or absence of NRG1β or PRL (whole cell lysates assessed by western blot analysis using the antibodies indicated at the *left*; *Mw* molecular weight marker) and **B** HC11 cells expressing shErbB3 or shScr serum and EGF-starved for 48 h in the presence of PRL, BKM120 (1 μM) or BSK805 (1 μM), with Annexin V-FITC added to cultures for the final 3 h, and the number of Annexin V^+^ cells counted (each *data point* shows the average of samples assessed in duplicate, relative to the average value of the number of Annexin V^+^ cells seen in untreated HC11-shScr cells; *midlines* are the average (± SD); *N* = 3) (PPTX 49017 kb)

## Abbreviations

aMEC: Alveolar mammary epithelial cell
d.p.c.: Days post coitus
EGFR: Epidermal growth factor receptor
*ErbB3*^*KO*^: ErbB3^FL/FL^ × WAPi-Cre
*ErbB3*^*WT*^: ErbB3^FL/FL^
JAK2: Janus kinase 2
L1: Lactation day 1
NRG: Neuregulin
PI3K: Phosphatidyl inositol-3 kinase
PIP3: Phosphoinositol-(3,4,5)-trisphosphate
PRL: Prolactin
RTK: Receptor tyrosine kinase
shErbB3: shRNA sequences against ErbB3
shScr: Scrambled shRNA sequences
STAT5: Signal transduction and activator of transcription
