# Supplementary material for: ErbB3 drives mammary epithelial survival and differentiation during pregnancy and lactation
Source: Breast Cancer Res. 2017 Sep 8;19:105. doi: 10.1186/s13058-017-0893-7 (PMC5591538; doi:10.1186/s13058-017-0893-7)
Supplement: Additional file 1: — is Figure S1 showing ErbB3 IHC of mammary glands harvested at 16.5 d.p.c. and L1, Figure S2 showing immunofluorescence for ErbB3 and Cytokeratin-8 (K8) in mammary glands harvested at 16.5 d.p.c., Figure S3 showing mammary glands harvested from 12–15-week-old mice at 16.5 d.p.c., L1 and L5, and stained using IHC for Ki67 (representative images, original magnification 400×), Figure S4 showing mammary glands harvested from 12–15-week-old mice at 16.5 d.p.c. and L1, assessed by TUNEL analysis in situ (representative images, original magnification 400×), Figure S5 showing P-Akt S473 IHC of mammary glands harvested from 12–15-week-old mice at 16.5 d.p.c., L1, and L5, and stained using IHC for P-Akt Serine 473 (representative images, original magnification 400×), Figure S6 showing HC11 cells treated with BKM120 (1 μM) or AZD6244 (1 μM) for 4 h: A western blot analysis using antibodies listed at the left (representative images, repeated three times) and B cell cultures stained with Annexin V-FITC to detect apoptotic cells, and Annexin V+ cells quantitated in digital images (experiments assessed in duplicate and repeated three times; data points are the average of the experimental duplicates; midlines are the average (± SD) of the biological replicates; Student’s unpaired t test), Figure S7 showing immunohistochemical detection of P-STAT5 A/B in mammary glands, Figure S8 showing HC11 cells expressing shErbB3 or shScr serum and EGF-starved for 24 h in the presence or absence of NRG1β (2 ng/ml), then stained with Annexin V-FITC to detect apoptotic cells, and Annexin V+ cells quantitated in digital images (experiments assessed in duplicate and repeated three times; data points are the average of the experimental duplicates; midlines are the average (± SD) of the biological replicates; Student’s unpaired t test), Figure S9 showing A HC11 cells expressing shErbB3 or shScr serum and EGF-starved for 24 h in the presence or absence of PRL, then whole RNA assessed by RT-qPCR for tran [file 13058_2017_893_MOESM1_ESM.pptx]

## Slide 1
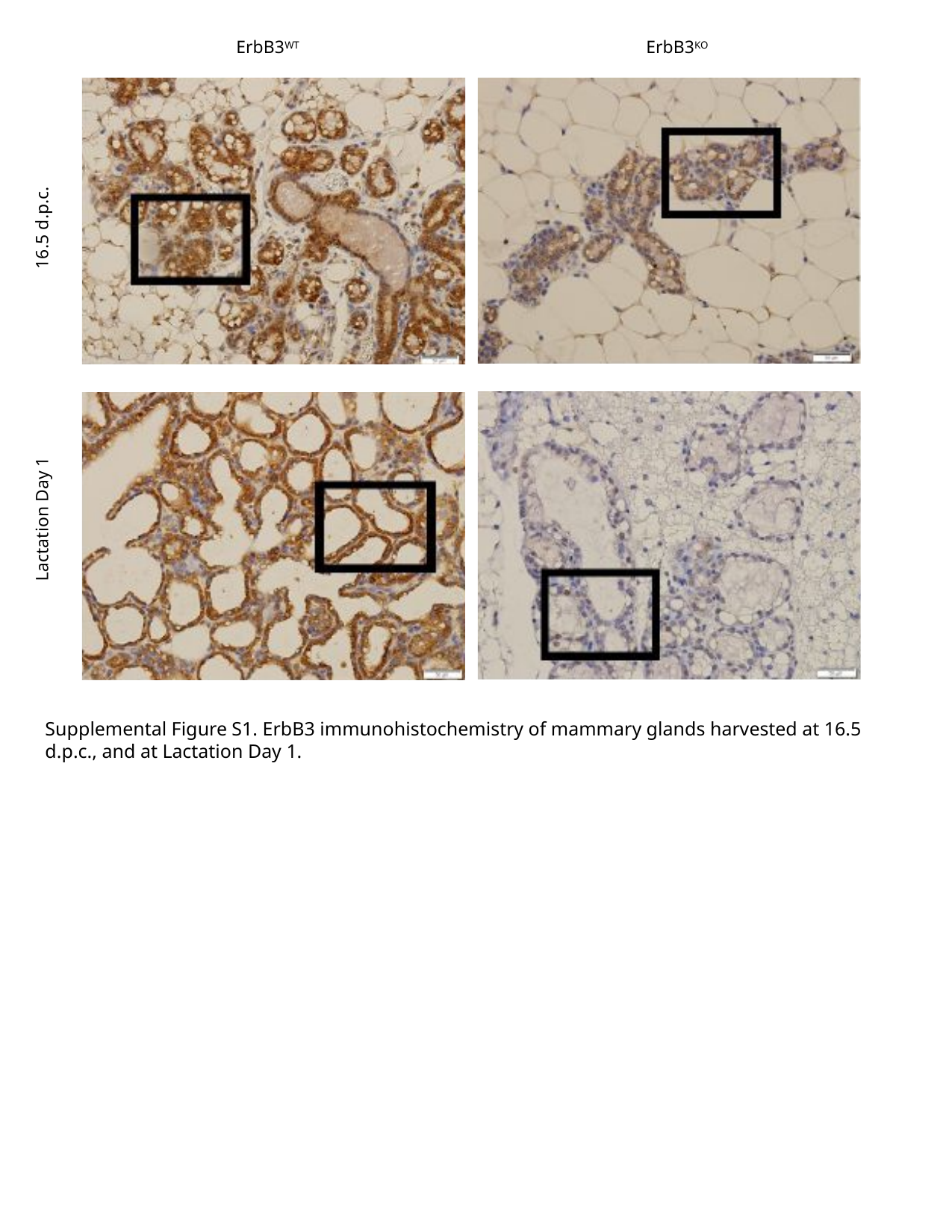

ErbB3WT
ErbB3KO
16.5 d.p.c.
Lactation Day 1
Supplemental Figure S1. ErbB3 immunohistochemistry of mammary glands harvested at 16.5 d.p.c., and at Lactation Day 1.

## Slide 2
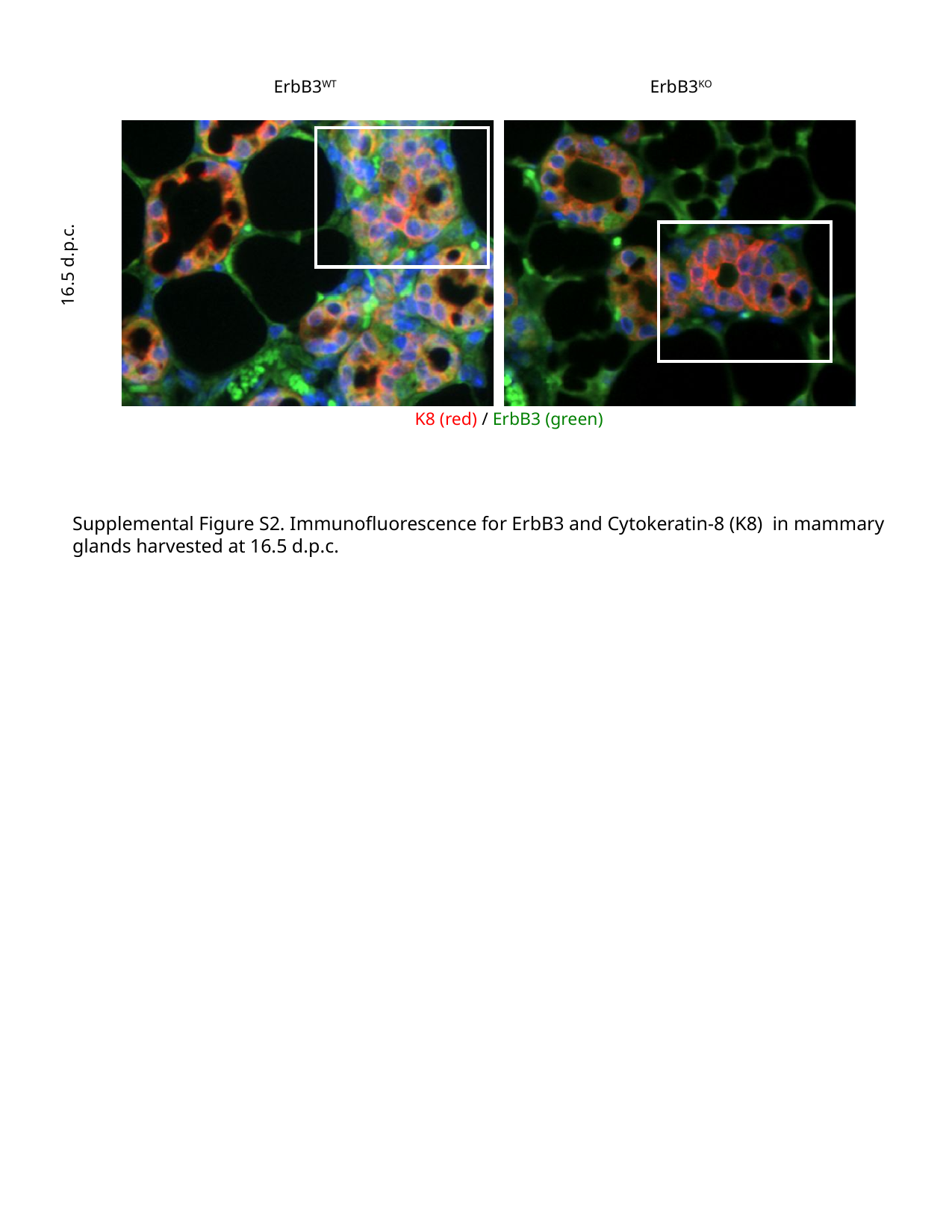

ErbB3WT
ErbB3KO
16.5 d.p.c.
K8 (red) / ErbB3 (green)
Supplemental Figure S2. Immunofluorescence for ErbB3 and Cytokeratin-8 (K8) in mammary glands harvested at 16.5 d.p.c.

## Slide 3
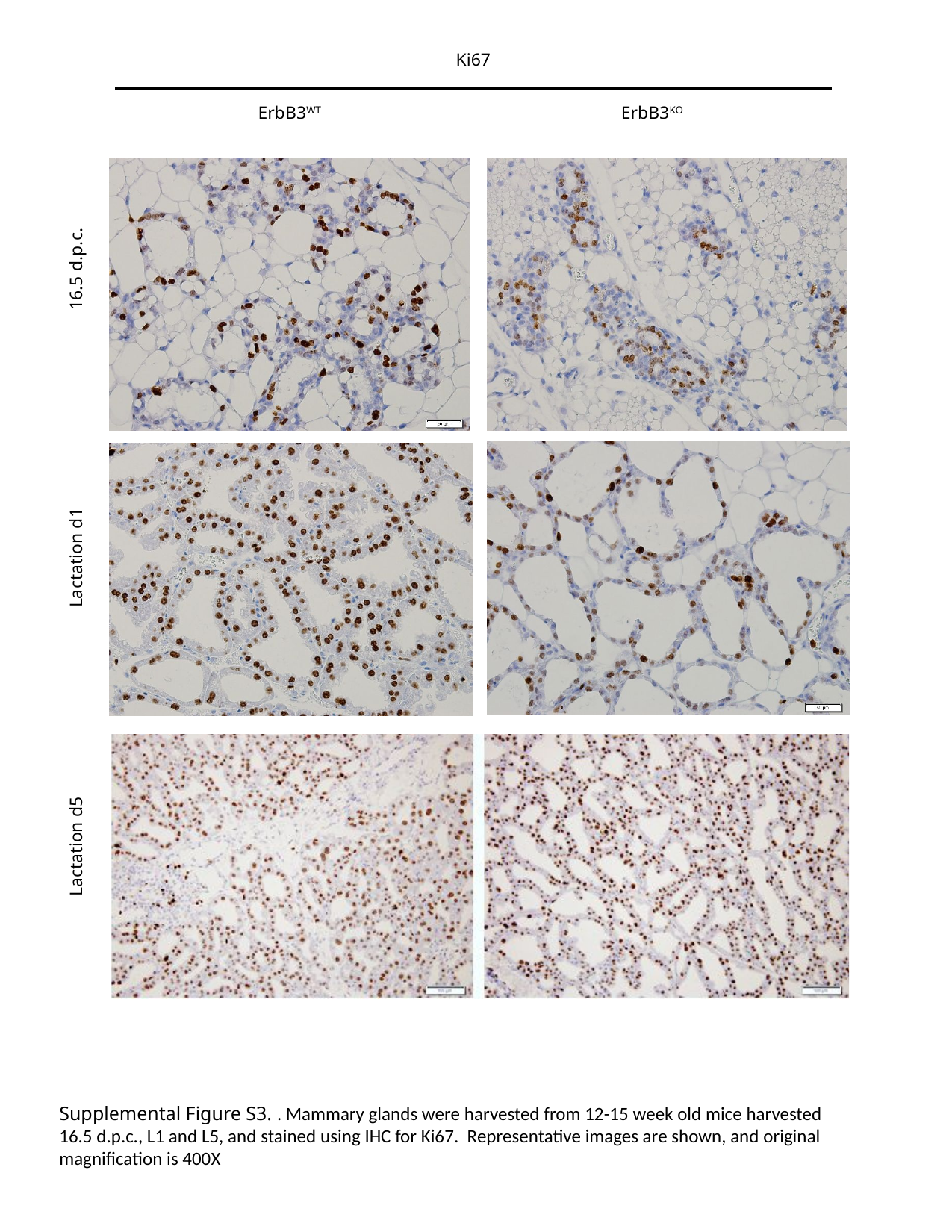

Ki67
ErbB3WT
ErbB3KO
16.5 d.p.c.
Lactation d1
Lactation d5
Supplemental Figure S3. . Mammary glands were harvested from 12-15 week old mice harvested 16.5 d.p.c., L1 and L5, and stained using IHC for Ki67. Representative images are shown, and original magnification is 400X

## Slide 4
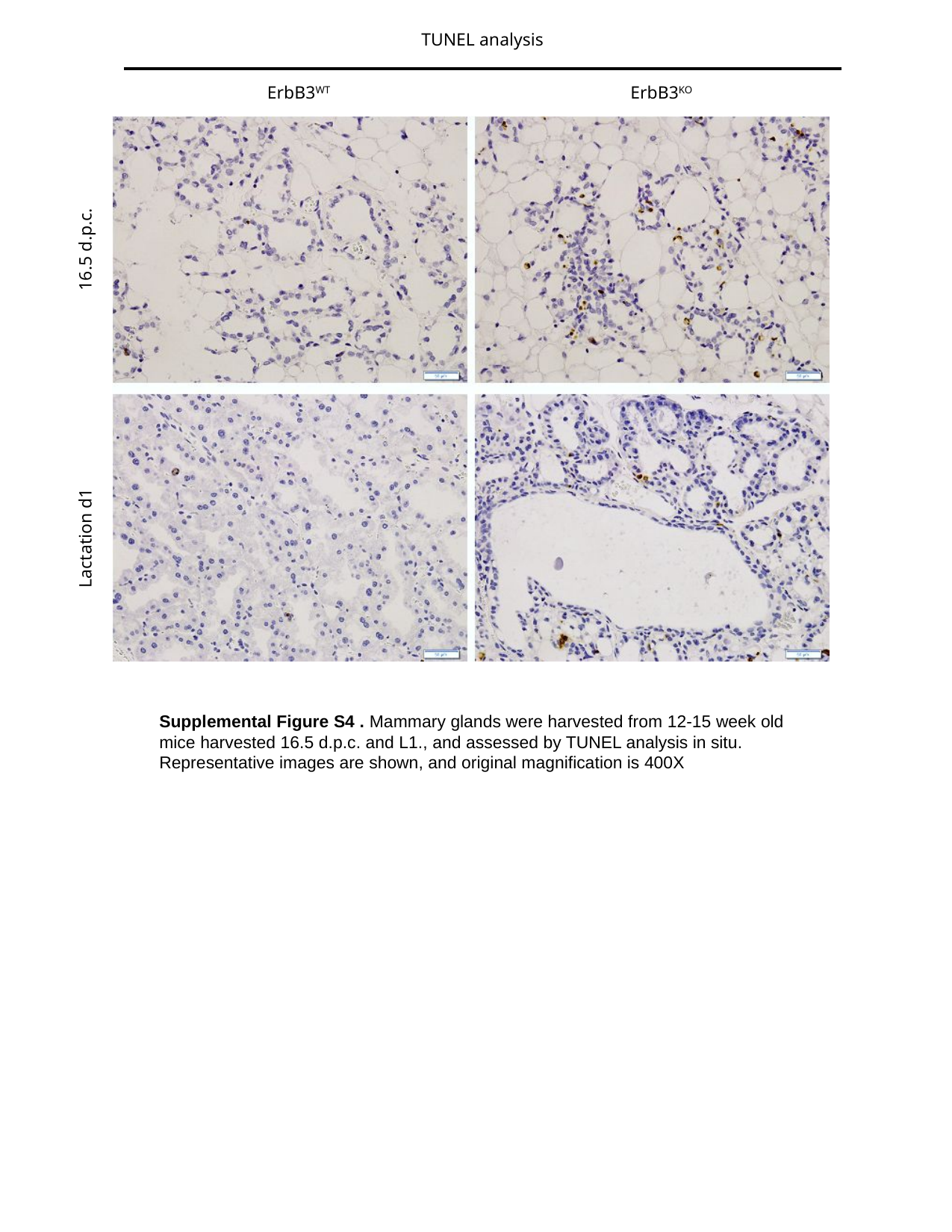

TUNEL analysis
ErbB3WT
ErbB3KO
16.5 d.p.c.
Lactation d1
Supplemental Figure S4 . Mammary glands were harvested from 12-15 week old mice harvested 16.5 d.p.c. and L1., and assessed by TUNEL analysis in situ. Representative images are shown, and original magnification is 400X

## Slide 5
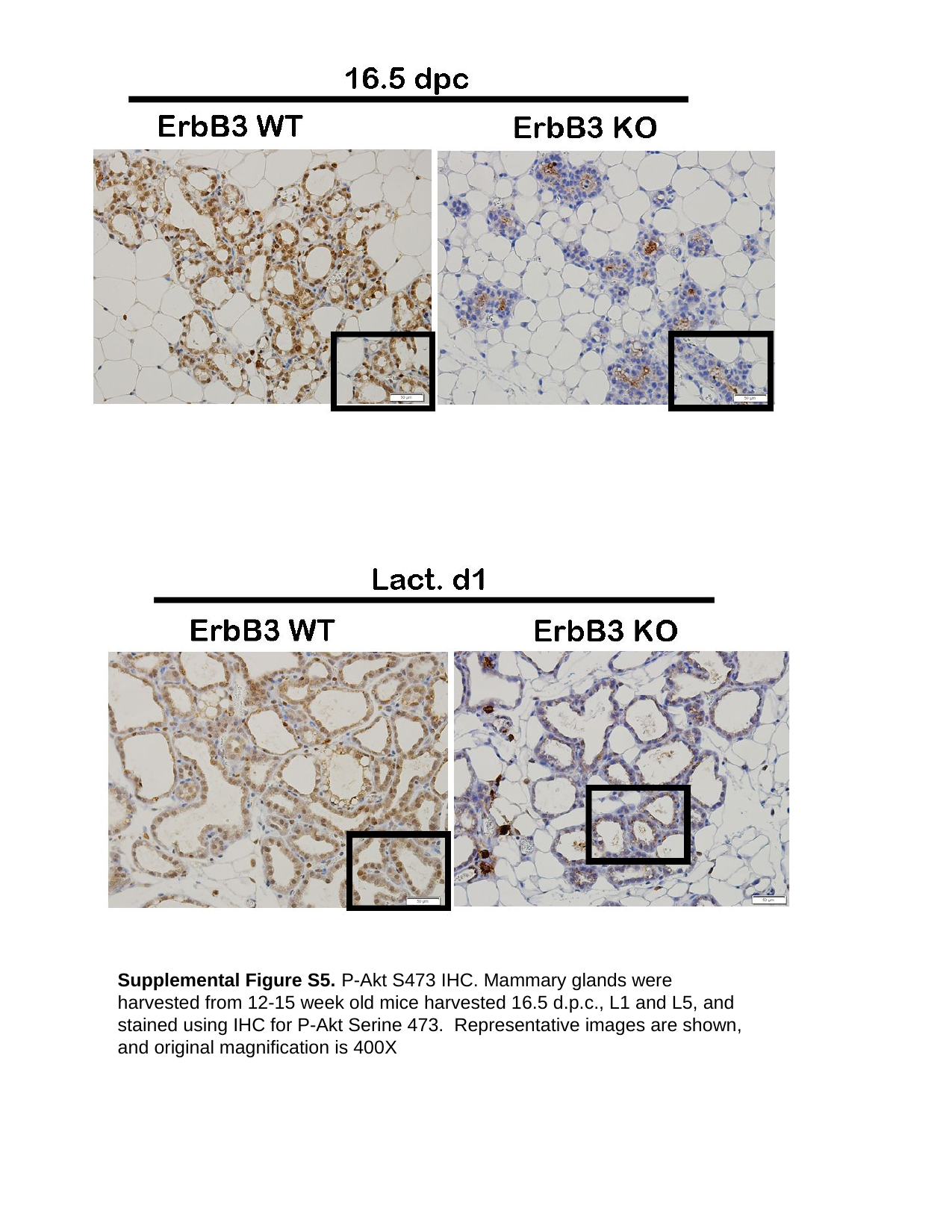

Supplemental Figure S5. P-Akt S473 IHC. Mammary glands were harvested from 12-15 week old mice harvested 16.5 d.p.c., L1 and L5, and stained using IHC for P-Akt Serine 473. Representative images are shown, and original magnification is 400X

## Slide 6
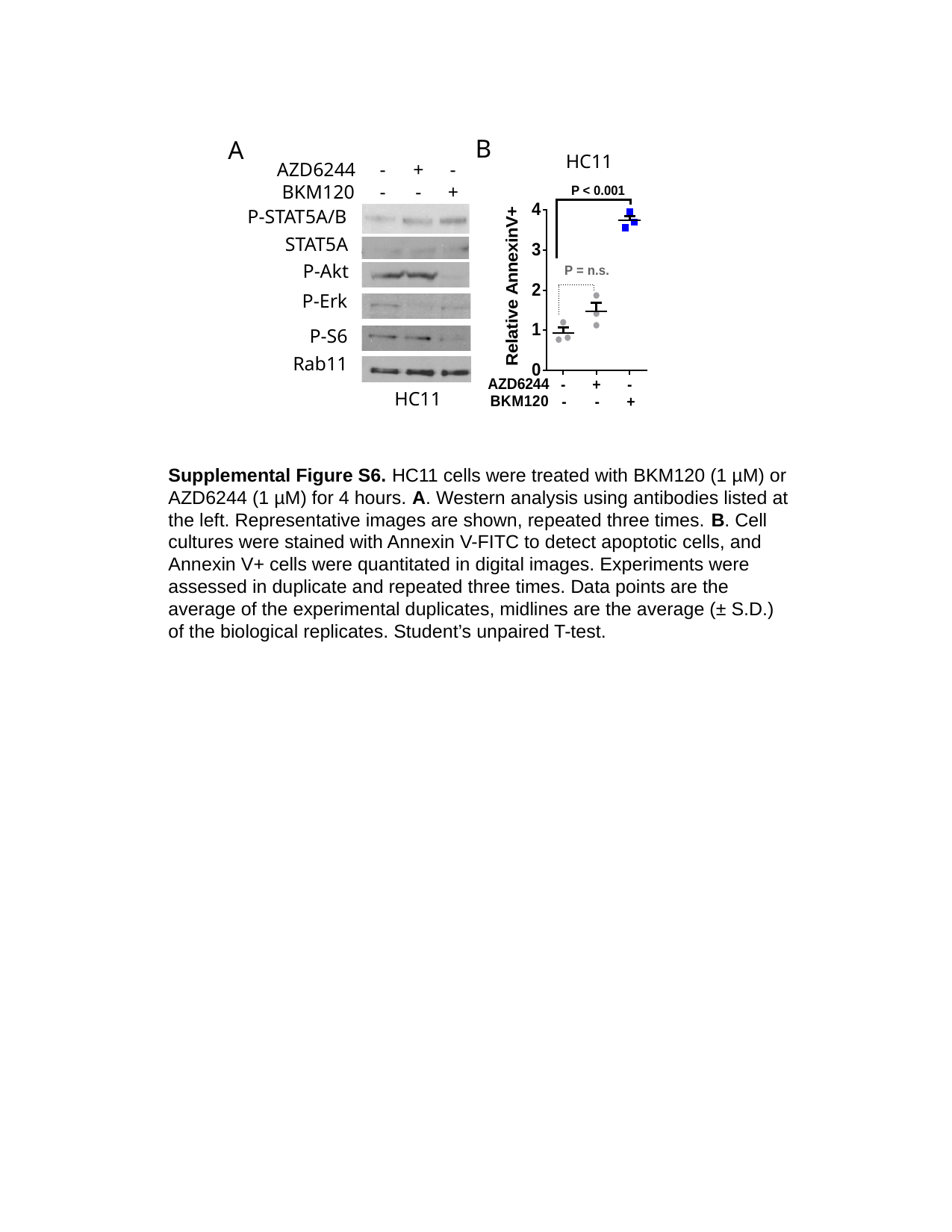

B
A
HC11
AZD6244
-
+
-
BKM120
-
-
+
P-STAT5A/B
STAT5A
P-Akt
P-Erk
P-S6
Rab11
HC11
Supplemental Figure S6. HC11 cells were treated with BKM120 (1 µM) or AZD6244 (1 µM) for 4 hours. A. Western analysis using antibodies listed at the left. Representative images are shown, repeated three times. B. Cell cultures were stained with Annexin V-FITC to detect apoptotic cells, and Annexin V+ cells were quantitated in digital images. Experiments were assessed in duplicate and repeated three times. Data points are the average of the experimental duplicates, midlines are the average (± S.D.) of the biological replicates. Student’s unpaired T-test.

## Slide 7
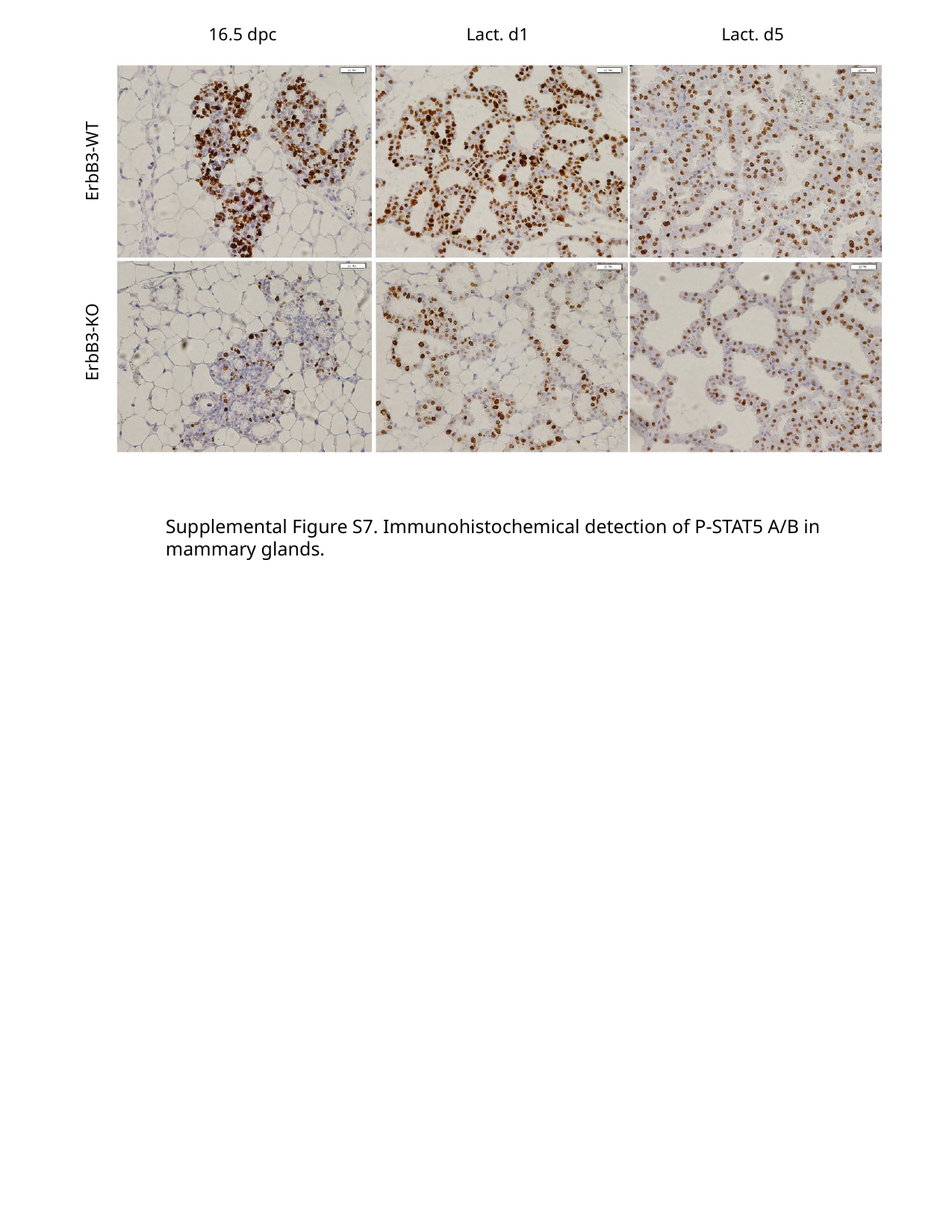

16.5 dpc
Lact. d1
Lact. d5
ErbB3-WT
ErbB3-KO
Supplemental Figure S7. Immunohistochemical detection of P-STAT5 A/B in mammary glands.

## Slide 8
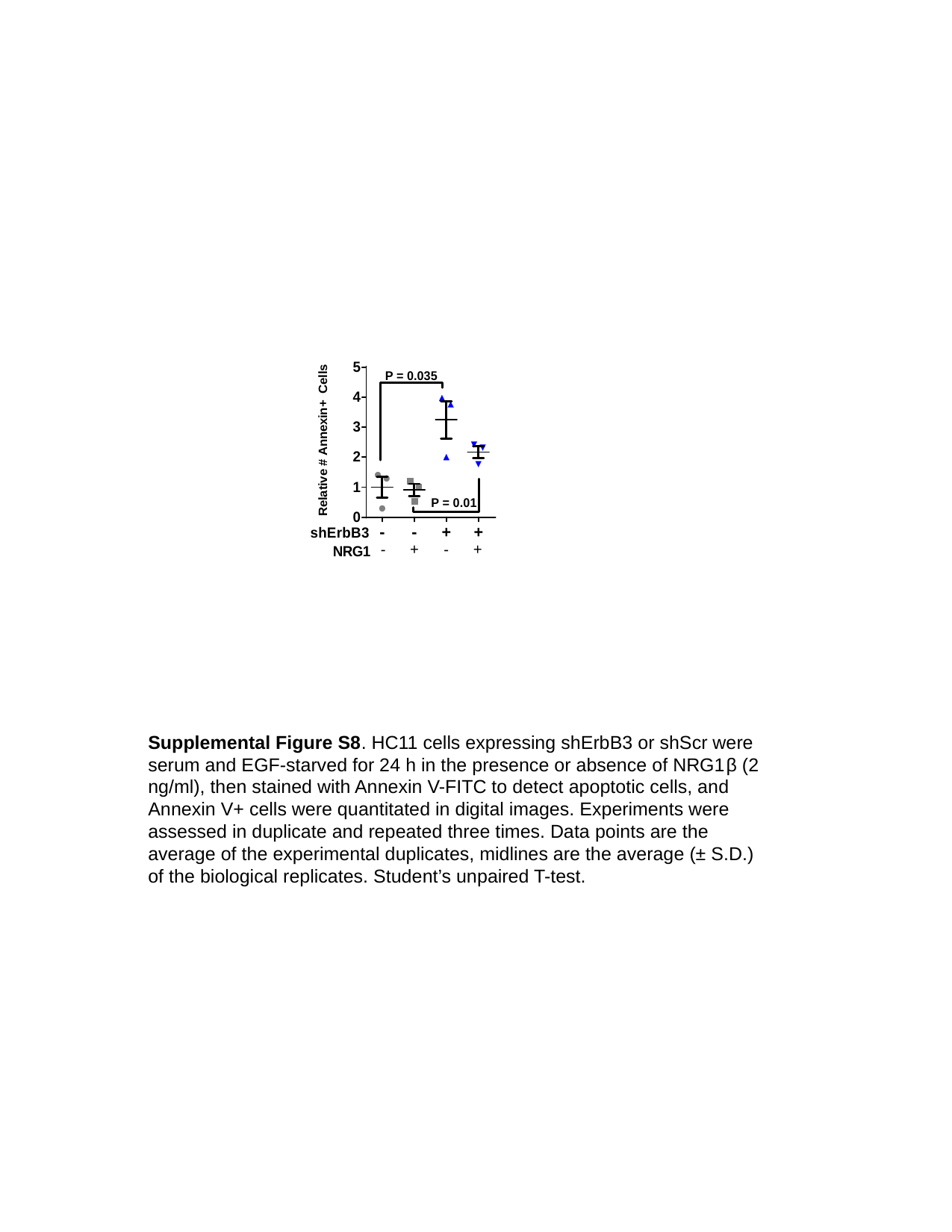

-
+
-
+
Supplemental Figure S8. HC11 cells expressing shErbB3 or shScr were serum and EGF-starved for 24 h in the presence or absence of NRG1β (2 ng/ml), then stained with Annexin V-FITC to detect apoptotic cells, and Annexin V+ cells were quantitated in digital images. Experiments were assessed in duplicate and repeated three times. Data points are the average of the experimental duplicates, midlines are the average (± S.D.) of the biological replicates. Student’s unpaired T-test.

## Slide 9
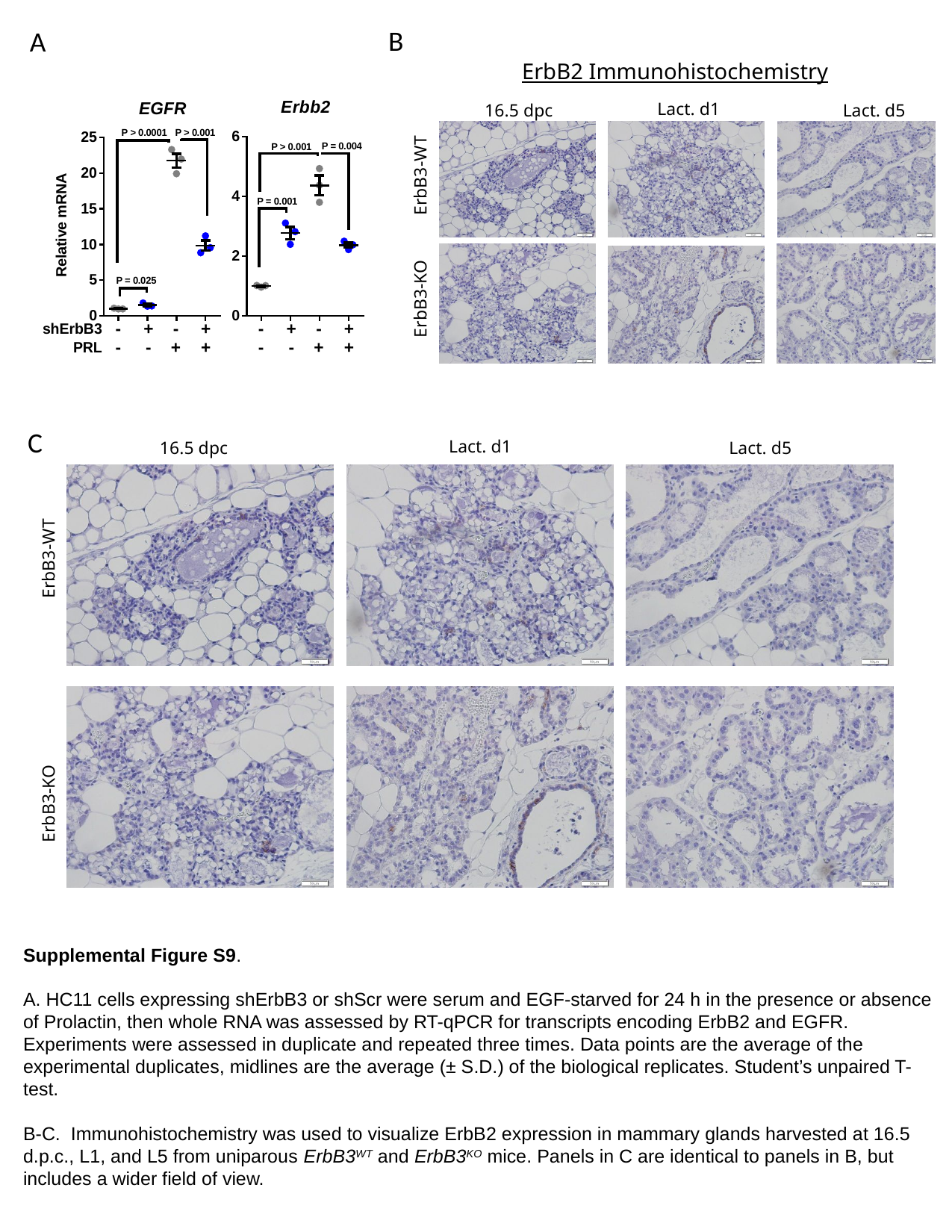

B
A
ErbB2 Immunohistochemistry
Lact. d1
16.5 dpc
Lact. d5
ErbB3-WT
ErbB3-KO
C
Lact. d1
16.5 dpc
Lact. d5
ErbB3-WT
ErbB3-KO
Supplemental Figure S9.
A. HC11 cells expressing shErbB3 or shScr were serum and EGF-starved for 24 h in the presence or absence of Prolactin, then whole RNA was assessed by RT-qPCR for transcripts encoding ErbB2 and EGFR. Experiments were assessed in duplicate and repeated three times. Data points are the average of the experimental duplicates, midlines are the average (± S.D.) of the biological replicates. Student’s unpaired T-test.
B-C. Immunohistochemistry was used to visualize ErbB2 expression in mammary glands harvested at 16.5 d.p.c., L1, and L5 from uniparous ErbB3WT and ErbB3KO mice. Panels in C are identical to panels in B, but includes a wider field of view.

## Slide 10
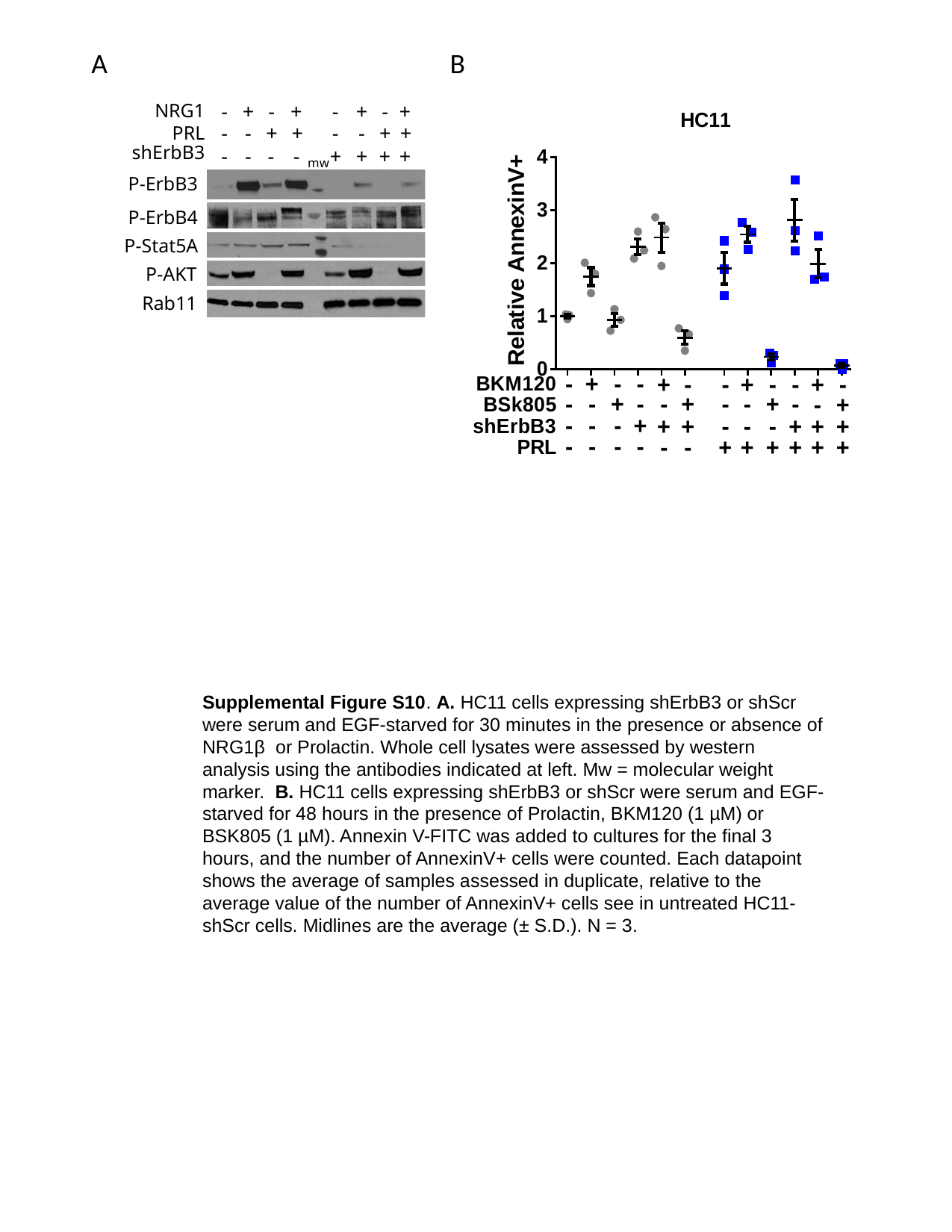

A
B
NRG1
-
+
-
+
-
+
-
+
-
-
+
+
-
-
+
+
PRL
shErbB3
-
-
-
-
+
+
+
+
mw
P-ErbB3
P-ErbB4
P-Stat5A
P-AKT
Rab11
Supplemental Figure S10. A. HC11 cells expressing shErbB3 or shScr were serum and EGF-starved for 30 minutes in the presence or absence of NRG1β or Prolactin. Whole cell lysates were assessed by western analysis using the antibodies indicated at left. Mw = molecular weight marker. B. HC11 cells expressing shErbB3 or shScr were serum and EGF-starved for 48 hours in the presence of Prolactin, BKM120 (1 µM) or BSK805 (1 µM). Annexin V-FITC was added to cultures for the final 3 hours, and the number of AnnexinV+ cells were counted. Each datapoint shows the average of samples assessed in duplicate, relative to the average value of the number of AnnexinV+ cells see in untreated HC11-shScr cells. Midlines are the average (± S.D.). N = 3.
